# CaMKII may regulate renal tubular epithelial cell apoptosis through YAP/NFAT2 in acute kidney injury mice

**DOI:** 10.1080/0886022X.2023.2172961

**Published:** 2023-01-31

**Authors:** Zongshun Huang, Yonghua Peng, Guibao Ke, Yun Xiao, Yaqi Chen

**Affiliations:** Department of Nephrology, First Affiliated Hospital of Guangzhou Medical University, Guangzhou, China

**Keywords:** Calcium/calmodulin-dependent protein kinase II, yes-associated protein, nuclear factor of activated T cells, cell apoptosis, acute kidney injury

## Abstract

**Aim:**

Renal tubular epithelial cell (RTEC) apoptosis is important in acute kidney injury (AKI). Calcium/calmodulin-dependent protein kinase II (CaMKII) plays an important role in cell apoptosis, but its potential role in AKI remains unknown.

**Methods:**

Using co-immunoprecipitation, immunofluorescence, immunohistochemistry, western blotting, flow cytometry, and cell transfection, this study aimed to verify whether CaMKII is involved in RTEC apoptosis and to explore the underlying mechanism.

**Results:**

We found that CaMKII was involved in RTEC apoptosis. In adriamycin-induced AKI mice, serum creatinine levels, cell apoptosis, CaMKII activity, and nuclear factor of activated T cells 2 (NFAT2) levels increased, whereas nuclear Yes-associated protein (YAP) expression decreased; inhibition of CaMKII activity reversed these changes. Phosphorylated CaMKII could bind to phosphorylated YAP in the cytoplasm and block it from entering the nucleus, thereby failing to inhibit NFAT2-mediated cell apoptosis. Sequestrated phosphorylated YAP in the RTEC cytoplasm was finally degraded by ubiquitination.

**Conclusion:**

CaMKII may regulate RTEC apoptosis through YAP/NFAT2 in AKI mice. CaMKII may be a potent molecular target for AKI treatment.

## 1. Introduction

Acute kidney injury (AKI) is a complicated syndrome characterized in the short term by the accumulation of metabolites and poisoning induced *via* a decline in kidney function. AKI is a global concern with a high incidence among patients across acute care settings [1,2]. Furthermore, AKI is associated with significant clinical consequences and increased healthcare costs [1–3]. However, the underlying mechanisms that cause AKI are still far from being fully understood, and AKI treatment is limited. Renal tubular epithelial cell (RTEC) apoptosis is one of the most important events in AKI. Therefore, to further exploring the underlying mechanism of RTEC apoptosis to identify treatment targets for AKI is of great significance.

Calcium/calmodulin-dependent protein kinase II (CaMKII) is a ubiquitously expressed multifunctional serine/threonine kinase that plays a critical role in cell apoptosis [4–6]. CaMKII pharmacological inhibition and genetic deletion protect against endoplasmic reticulum stress-induced cardiomyocyte apoptosis [7]. Furthermore, CaMKII overexpression enhances iohexol-induced mitochondrial damage and RTEC apoptosis [4]. However, it remains unclear whether CaMKII participates in the pathogenesis of RTEC apoptosis in AKI.

The nuclear factor of activated T cells (NFAT) is a Ca^2+^-dependent transcription factor that functions as a calcineurin substrate. Studies have shown that nuclear NFAT2 is significantly increased in high glucose-injured podocytes, which is reversed by calcineurin/NFAT2 signal inhibition [8,9]. Furthermore, CaMK is known to promote NFAT expression [10]. These findings indicate that CaMKII and NFAT2 may participate in the pathogenesis of RTEC apoptosis.

Yes-associated protein (YAP) is an important member of the Hippo pathway and plays a critical role in cell survival and proliferation [11,12]. Under normal conditions, dephosphorylated YAP locates the nucleus and functions as a transcriptional co-activator, interacting with TEA domain family member transcription factors to regulate target gene expression [13]. Phosphorylated YAP (pYAP) is sequestered in the cytoplasm, remains inactive, and is degraded by ubiquitination [14]. Our previous studies showed that nuclear YAP is remarkably reduced in adriamycin (ADR)-injured podocytes. Knocking down YAP expression using specific siRNA induces podocyte apoptosis, and knocking down NFAT2 expression ameliorates YAP-siRNA-mediated podocyte apoptosis [15–17]. Moreover, knocking down YAP expression using specific siRNA inhibits RTEC proliferation [18]. These findings indicate that YAP and NFAT2 may also play important roles in RTEC apoptosis.

Therefore, the aim of this study was to verify whether CaMKII is involved in RTEC apoptosis and to explore the underlying mechanism. The results of this study provide new insights and potential targets for ameliorating RTEC apoptosis in AKI.

## 2. Materials and methods

### 2.1. Cell culture

The RTEC line HK-2 (ATCC) was cultured at 37 °C in RPMI-1640 medium (Gibco BRL, Gaithersburg, MD, USA) supplemented with 10% fetal bovine serum (FBS, Gibco BRL, USA) before maturation. Approximately 1 x 10^6^ matured cells were treated with ADR (0.0625 μg/mL; doxorubicin hydrochloride; Sigma, St Louis, MO, USA), KN-93 phosphate (K93, a specific inhibitor of CaMKII, 2 μM; Selleck Chemicals, Houston, TX, USA), or calmodulin (CaM, an activator of CaMKII, 5 μM; Santa Cruz, USA) for 24 h before harvest.

### 2.2. Transfection of siRNAs and plasmids

Small-interfering RNAs (siRNAs) for control, YAP, and NFAT2 were synthesized by RiboBio Co. Ltd. (Guangzhou, China). The sequences of YAP- and NFAT2-siRNAs were as follows: 5′-CGAGAUGAGAGCACAGACA-3′ and 5′-GCCATAACTTTCTGCAAGA-3′. siRNAs and overexpressed YAP plasmids were transfected as described in our previous studies [16,17]. In addition, wild-type YAP and YAP S112A mutation plasmids (overexpressed YAP plasmids, CS-Mm06093-Lv128) were purchased from GeneCopoeia Co. Ltd. (Guangdong, China) and synthesized according to the protocol of the Lenti-Pac™ HIV Expression Packing Kit (HPK-LvTR-20, GeneCopoeia, China). Approximately 1 x 10^6^ matured HK-2 cells were infected with siRNA or lentivirus particles for 24 h before harvesting.

### 2.3. Mice

A total of 18 female BALB/c mice (6 to 8 weeks old) were purchased from the Model Animal Research Center of Nanfang Medical University (Guangdong, China). All animal experiments were performed according to the ARRIVE guidelines [19] and approved by the Institutional Animal Care and Use Committee of Guangzhou Medical University (Approval No: 2017-224; Guangdong, China). Mice were divided into three groups with six mice in each group: normal control group (Con), adriamycin-injured group (ADR), and adriamycin and K93 (KN-93 phosphate, Selleck Chemicals)-treated group (ADR + K93). A total of 12 mice were injected once with ADR (12 mg/kg body weight) *via* the tail vein on day 0 [16,20], and 6 control mice were injected with an equal volume of saline solution. A total of 6 ADR mice were intraperitoneally injected with K93 at a dose of 0.08 mg/kg body weight on days 1, 3, and 5. Finally, mice were anesthetized with ketamine (70 mg/kg, i.p.) before euthanasia on day 7. Then, arterial blood was collected for ELISA, and kidney tissues were collected for western blotting, HE staining, immumohistochemical staining, and TUNEL staining analyses. A mouse creatinine kit (Cayman Chemical, USA) was used to measure serum creatinine levels in mice according to the manufacturer’s instructions.

### 2.4. Western blotting

Cytoplasmic, nuclear, and whole proteins from RTEC or kidney tissue were prepared according to the manufacturer’s protocol (Nanjing KeyGEN Biotech, China), as described in our previous studies [16,17]. Cell lysates containing 30 μg protein were separated on 10% sodium dodecyl sulfate–polyacrylamide gels and then transferred to polyvinylidene fluoride membranes. After blocking with 5% skim milk for 1 h at room temperature, membranes were incubated overnight at 4 °C with the following primary antibodies: rabbit anti-phospho-CaMKII (Cell Signaling Technology, USA; 1:1000), rabbit anti-CaMKII (Santa Cruz, USA; 1:500), rabbit anti-YAP (Cell Signaling Technology; 1:1000), rabbit anti-NFAT2 (Abcam, USA; 1:1000), rabbit anti-Bcl-2 (Cell Signaling Technology; 1:1000), rabbit anti-Bax (Cell Signaling Technology; 1:1000), rabbit anti-GAPDH (Bioworld Technology, China; 1:10000), and rabbit anti-Histone (Cell Signaling Technology; 1:3000). After incubation with anti-rabbit IgG (Jackson Immuno Research, USA, 1:4000) at room temperature for 1 h, membranes were treated with ECL reagents (Pierce Chemical, IN, USA), and then exposed to X-ray film (Kodak, USA). Quantitative densitometric analysis of autoradiographic bands was conducted using BandScan software. Relative protein expression is presented as the ratio of target protein band intensity to GAPDH or Histone (nuclear fractions) band intensity.

### 2.5. Immunoprecipitation

Cell lysates containing cytoplasmic or nuclear proteins from RTEC were prepared according to the manufacturer’s protocol (Nanjing KeyGEN Biotech). Immunoprecipitation was performed according to the Pierce Co-Immunoprecipitation Kit protocol (Pierce, USA). Briefly, 500 μl cell lysates were precleared with 20 μl protein A/G agarose beads (Santa Cruz, USA), and then incubated overnight at 4 °C with 1 μl of the following primary antibodies: rabbit anti-pCaMKII, rabbit anti-YAP, and rabbit anti-HA-IgG as a negative control. After incubation with 15 μl protein A/G agarose beads (Santa Cruz, USA) at 4 °C for 2 h, the cell lysates were centrifugated. The precipitates were collected and subjected to western blotting as described above. When blotting pYAP, a pCaMKII antibody was used to pull down the combination of cytoplasmic pYAP and pCaMKII. Similarly, when blotting nuclear NFAT2, a YAP antibody was used to pull down the combination of nuclear NFAT2 and YAP.

### 2.6. Ubiquitinated pYAP analysis

Cell lysates containing only cytoplasmic protein from RTEC were prepared according to the manufacturer’s protocol (Nanjing KeyGEN Biotech). Immunoprecipitation was carried out as described above. Briefly, 500 μl cell lysates were precleared with 20 μl protein A/G agarose beads, and then incubated overnight at 4 °C with 1 μl of rabbit anti-pYAP antibody. After incubation with 15 μl protein A/G agarose beads at 4 °C for 2 h, cell lysates were centrifugated, and the precipitates were collected for western blotting. Immunoprecipitated protein was immunoblotted with the anti-ubiquitin antibody (Cell Signaling Technology, USA).

### 2.7. Annexin V and propidium iodide staining assay

As described in our previous studies [16,17], apoptotic RTECs were detected *via* an Annexin V (conjugated with FITC)/propidium iodide (PI) apoptosis detection kit (Nanjing KeyGEN Biotech), according to the manufacturer’s protocol. Briefly, after resuspension with 500 μl of binding buffer, cultured RTECs were incubated with 5 μl of Annexin V and PI in the dark for 10 min, and then cell fluorescence was analyzed using a Cell Lab QuantaTM SC Flow cytometer (Beckman Colter, Inc, USA). Apoptotic RTECs were positive for Annexin V-FITC.

### 2.8. Immunofluorescent staining and TUNEL staining

The procedure for immunofluorescent and TUNEL staining of RTECs was similar to that used in our previous studies [16,17]. Briefly, RTECs cultured on cover slides in 12-well plates or frozen cryostat sections from mouse renal cortex were fixed with 4% paraformaldehyde at RT for 20 min. After permeabilization with 0.1% Triton X-100 and blocking with 5% bovine serum albumin, cells and sections were incubated overnight at 4 °C with the following primary antibodies: rabbit anti-phospho-CaMKII (Cell Signaling Technology; 1:100), goat anti-YAP (Cell Signaling Technology; 1:100), and rabbit anti-NFAT2 (Abcam; 1:100). Cultured RTECs or sections were washed with phosphate buffer saline (PBS), and then incubated for 1 h at RT in the dark with secondary antibodies (goat anti-rabbit Alexa Fluor 555, Cell Signaling Technology, 1:200; FITC-donkey anti-goat IgG 488, Protein Tech Group, Inc, Rosemont, IL, USA, 1:200), and stained with DAPI (Sigma, USA) for 5 min. Apoptotic cells in sections were detected *via* a TUNEL kit (Roche Molecular Biochemicals, Germany). Photomicrographs were obtained *via* confocal laser scanning microscopy (LeCSM, Zeiss KS 400; Postfach, Germany) and analyzed by Image-Pro Plus 6.0 (Media Cybernetics, Georgia Avenue, MD, USA) for quantification [16,17]. Eighteen random fluorescence sights from six cover slides or sections in each group were selected to conduct the quantitative analysis. Two investigators who were blinded to the original samples analyzed all images.

### 2.9. Immunohistochemical staining

Paraffin-embedded kidney tissue sections were deparaffinized with dimethylbenzene and hydrated with ethanol and water. The sections were placed in 0.01 M citrate buffer and heated for 20 min at 95 °C in a water bath. After cooling to room temperature, slides were immersed in 3% H_2_O_2_ to quench endogenous peroxidase activity. Slides were then washed with PBS and incubated with rabbit anti-phospho-CaMKII (Cell Signaling Technology; 1:100), rabbit anti-YAP (Cell Signaling Technology; 1:100), and rabbit anti-NFAT2 (Abcam, USA; 1:100) overnight at 4 °C. After washing with PBS, the slides were incubated for 1 h at room temperature with a horseradish peroxidase-labeled secondary antibody and then washed. Immunoperoxidase staining was performed using a Ready-to-use Immunohistochemical SP Kit (Nanjing KeyGEN Biotech, China) according to the manufacturer’s instructions. The slides were counterstained with hematoxylin and mounted before examination by light microscopy. We selected 18 random sites from 6 sections in each group to conduct quantitative analysis *via* Image-Pro Plus 6.0 [16,21], (Media Cybernetics, Georgia Avenue, MD, USA). Two investigators who were blinded to the original samples analyzed all images.

### 2.10. Statistical analysis

All values are expressed as mean ± standard error (SE). Statistical data were analyzed using SPSS Ver. 22.0 (SPSS, Inc., Chicago, IL, USA). All experimental observations were repeated at least thrice. Data from multiple groups were analyzed using Bonferroni’s or Tamhane’s T2 tests. Comparisons between two groups were conducted using Student’s *t*-test. Statistical significance was set at *p* < 0.05.

## 3. Results

### 3.1. CaMKII activity increased in ADR-injured RTECs

**Figure 1.**
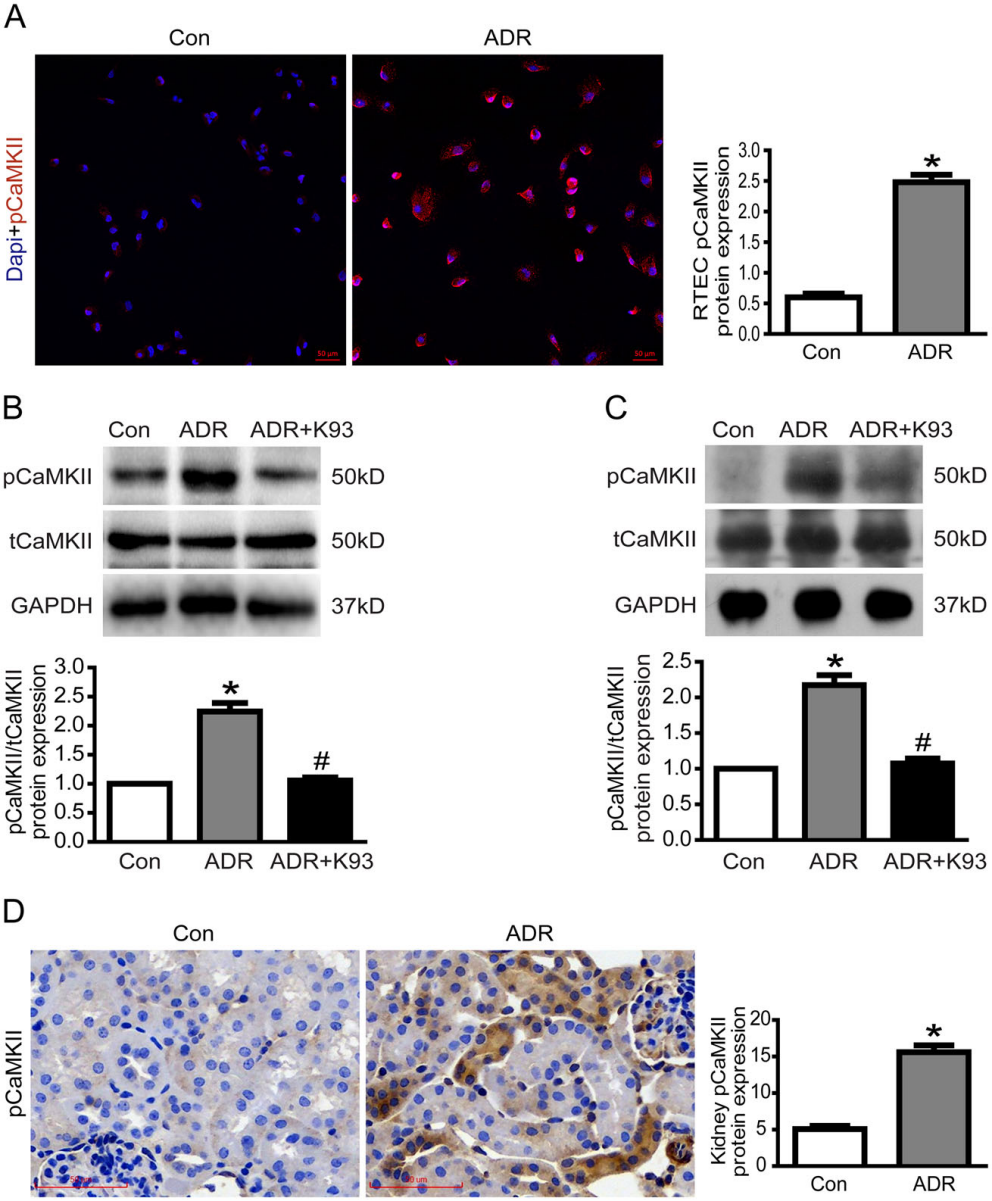
CaMKII activity was increased in ADR-injured RTECs. (A) Representative micrographs of dual-color fluorescence staining for pCaMKII (red) and DAPI (blue) from Con and ADR culture RTECs. Magnification ×200, scale bar = 50 μm. (B) Western blotting showed that the CaMKII activity (the protein expression of pCaMKII/tCaMKII) increased in ADR-injured culture RTECs, and this was prevented by using K93 (CaMKII activity inhibitor; *p* < 0.01). (C) CaMKII activity increased in kidney tissue cells of ADR-treated mice, and this was prevented by using K93 (*p* < 0.01). (D) Representative micrographs of immunohistochemical staining for pCaMKII (brown) in kidney sections. Magnification ×400, scale bar = 50 μm. Mean ± SE, *n* = 6. *p < 0.01 vs. Con. #*p* < 0.01 vs. ADR. There was no statistically significant difference between the Con and ADR + K93 groups.

Several studies [6,7,22,23] have shown that the activity of CaMKII markedly increases in different cell types under undesired exposure, and we speculated that CaMKII activity might also increase in ADR-injured RTECs. Thus, we measured CaMKII activity (pCaMKII/tCaMKII) in ADR-injured RTECs *in vitro* and *in vivo*. As shown in Figure 1(A), immunofluorescent staining showed that cytoplasmic pCaMKII expression increased in ADR-injured cultured RTECs compared with control cultured RTECs. Moreover, western blotting results showed that CaMKII activity increased significantly in ADR-injured RTECs (*p* < 0.01). K93 (KN-93 phosphate) inhibited CaMKII activity in ADR-injured RTECs (*p* < 0.01). The activity of CaMKII was 100%, 224.3 ± 14.8%, and 105.6 ± 5.3% in the control, ADR, and ADR + K93 groups, respectively (Figure 1(B)). Additionally, western blotting results (Figure 1(C)) revealed that CaMKII activity increased significantly in kidney tissue cells of ADR-treated mice (*p* < 0.01). Inhibition of CaMKII activity using K93 prevented this increase in activity (*p* < 0.01). The activity of CaMKII was 100%, 217.3 ± 14.0%, and 107.5 ± 6.7% in the control, ADR, and ADR + K93 groups, respectively. Furthermore, immunohistochemical staining results (Figure 1(D)) revealed that cytoplasmic pCaMKII increased significantly in RTECs of ADR-treated mice compared with controls.

### 3.2. Inhibition of CaMKII activity ameliorated ADR-induced RTEC apoptosis and kidney injury

**Figure 2.**
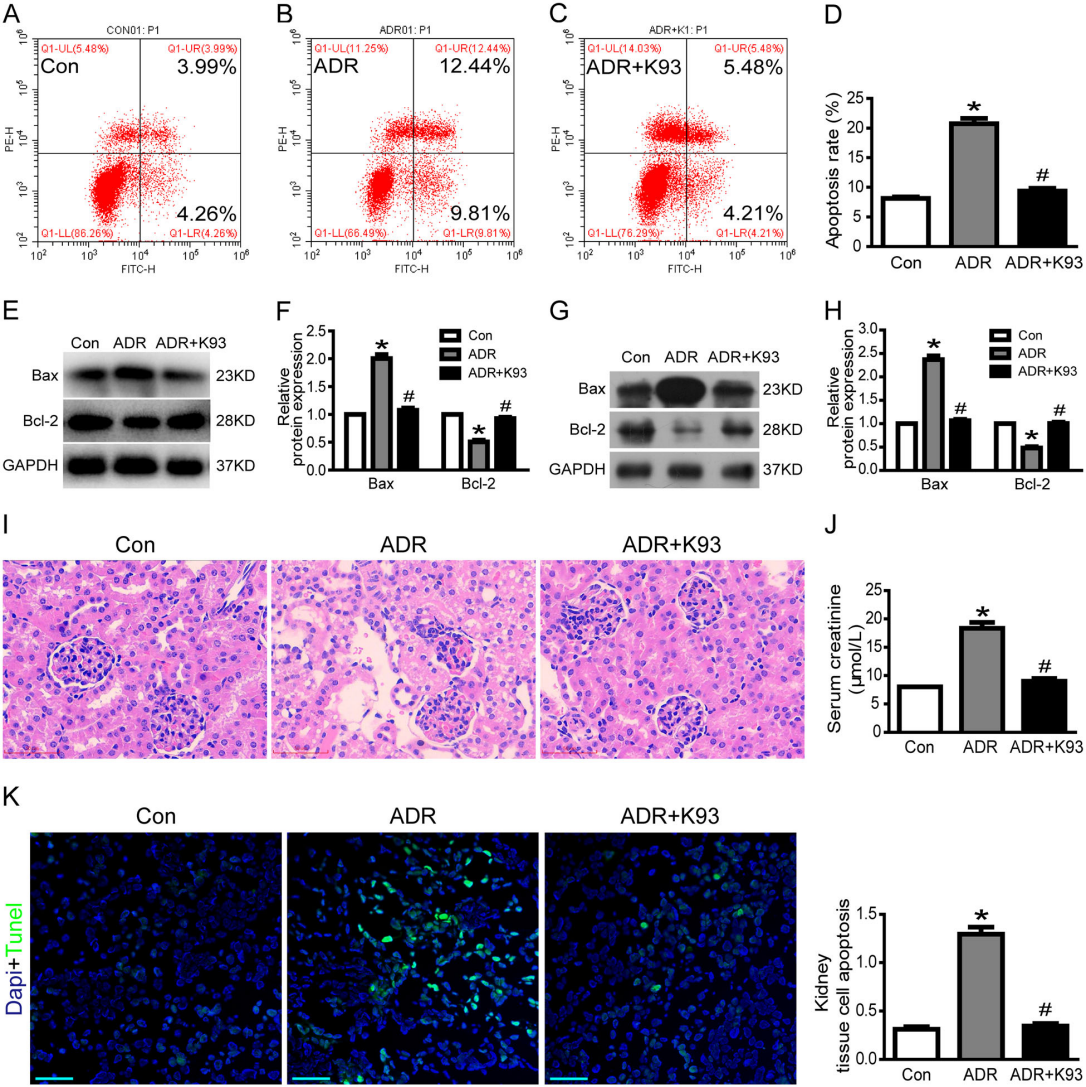
Inhibition of CaMKII activity ameliorated ADR-induced RTEC apoptosis and kidney injury. (A–D) Culture RTECs were stained with Annexin V/PI for flow cytometry analysis. Cell apoptosis rate increased significantly in ADR-injured RTECs compared with that in the controls (*p* < 0.01). However, the cell apoptosis rate was ameliorated by using K93 (CaMKII activity inhibitor) in ADR-injured RTECs (*p* < 0.01). (E–F) Western blotting showed that Bax protein expression increased while Bcl-2 decreased in ADR-injured RTECs (*p* < 0.01). However, inhibition of CaMKII activity *via* K93 prevented these changes (*p* < 0.01). (G–H) Bax protein expression increased while Bcl-2 decreased in kidney tissue cells of ADR-treated mice (*p* < 0.01). However, inhibition of CaMKII activity *via* K93 prevented these changes (*p* < 0.01). (I) HE staining of ADR-treated mice showing damaged epithelial cells in renal tubules. Inhibition of CaMKII activity *via* K93 ameliorated this damage. Magnification ×400, scale bar = 50 μm. (J) Serum creatinine levels increased in ADR-treated mice (*p* < 0.01). However, serum creatinine level was almost normal after inhibiting CaMKII activity using K93 in ADR-treated mice (*p* < 0.01). (K) Kidney tissue cell apoptosis was examined *via* the TUNEL assay. Apoptotic cells (sky blue-stained) increased in ADR-treated mice, but K93 treatment prevented apoptosis. Magnification ×200, scale bar = 50 μm. Mean ± SE, *n* = 6. *p < 0.01 vs. Con. #*p* < 0.01 vs. ADR. There was no statistically significant difference between the Con and ADR + K93 groups.

Our previous studies [16,17] showed that apoptosis increases significantly in ADR-injured podocytes both *in vitro* and *in vivo*. Therefore, we speculated that apoptosis might be increased in ADR-injured RTECs both *in vitro* and *in vivo*. As expected, the apoptosis rate increased significantly in ADR-injured RTECs compared with control RTECs *in vitro* (*p* < 0.01). Inhibition of CaMKII activity using K93 ameliorated ADR-induced RTEC apoptosis (*p* < 0.01). Cell apoptosis rates were 8.1% ± 0.3%, 20.7% ± 0.9%, and 9.4% ± 0.5% in the control, ADR-injured, and ADR + K93 group RTECs, respectively (Figure 2(A–D)). Furthermore, the protein expression of Bax, a well-recognized indicator of apoptosis, increased significantly, whereas the protein expression of Bcl-2, an indicator of anti-apoptosis, decreased significantly in ADR-injured RTECs and kidney tissue cells of ADR-treated mice (*p* < 0.01). Inhibition of CaMKII activity using K93 prevented these changes (*p* < 0.01; Figure 2(E–H)). Consequently, in ADR-treated mice, HE staining showed epithelial cell damage in a few renal tubules. Inhibition of CaMKII activity *via* K93 ameliorated this damage (Figure 2(I)). Serum creatinine levels also increased in ADR-treated mice (*p* < 0.01). However, these levels returned to almost normal after inhibiting CaMKII activity with K93 in ADR-treated mice (*p* < 0.01; Figure 2(J)). Kidney tissue cell apoptosis was examined *via* a TUNEL assay, which demonstrated that apoptotic cells increased in ADR-treated mice, but K93 treatment prevented apoptosis (*p* < 0.01; Figure 2(K)). These data show that inhibition of CaMKII activity ameliorated ADR-induced RTEC apoptosis and kidney injury, and CaMKII played an important role in RTEC apoptosis and AKI.

### 3.3. Inhibition of CaMKII activity prevented ADR-induced nuclear YAP decrease

**Figure 3.**
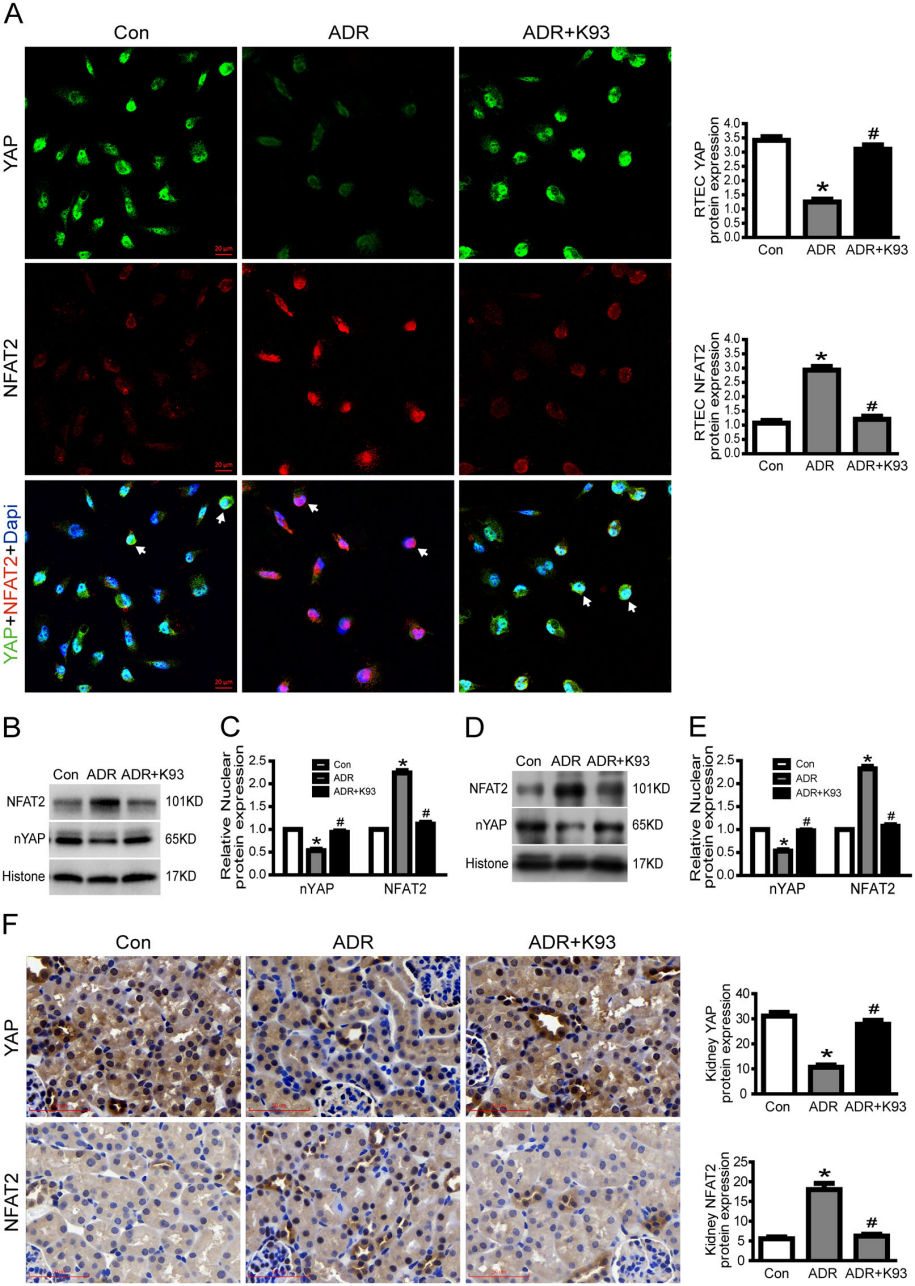
Inhibition of CaMKII activity prevented ADR-induced decrease in nuclear YAP and increase in nuclear NFAT2. (A) Typical confocal images of culture RTECs showing expression of YAP (green), NFAT2 (red), and DAPI-stained nuclei (blue). White arrows point to typical overlap of YAP and NFAT2 protein in the RTEC nucleus. Magnification ×400, scale bar = 20 μm. (B–C) Western blotting shows that nuclear protein expression of YAP decreased significantly whereas NFAT2 increased significantly in ADR-injured RTECs (*p* < 0.05). However, inhibition of CaMKII activity using K93 reversed these changes (*p* < 0.05). (D–E) Nuclear protein expression of YAP decreased significantly whereas NFAT2 increased significantly in kidney tissue cells of ADR-treated mice (*p* < 0.05), and inhibition of CaMKII activity reversed the changes (*p* < 0.05). (F) Representative micrographs of immunohistochemical staining for YAP and NFAT2 (brown) from kidney sections. Magnification ×400, scale bar = 50 μm. Mean ± SE, *n* = 6. *p < 0.05 vs. Con. #*p* < 0.05 vs. ADR. There was no statistically significant difference between the Con and ADR + K93 groups.

As our previous studies [16,17] showed that YAP expression is remarkably reduced in ADR-injured podocytes, we investigated the expression of YAP in ADR-injured RTECs *in vitro* and *in vivo* to explore the underlying mechanism of YAP in RTEC apoptosis. We used confocal microscopy to examine the localization of YAP and western blotting to analyze nuclear YAP protein expression. As shown in Figure 3(A–C), the results of immunofluorescence analysis and western blotting indicate that the nuclear protein expression of YAP decreased markedly in ADR-injured cultured RTECs. Surprisingly, inhibition of CaMKII activity using K93 prevented the ADR-induced decrease in YAP expression. In addition, the western blotting results show that the nuclear protein expression of YAP decreased significantly in kidney tissue cells of ADR-treated mice (*p* < 0.05), and inhibition of CaMKII activity using K93 prevented this change (*p* < 0.05; Figure 3(D–E)). Furthermore, immunohistochemical staining results indicated that nuclear YAP decreased significantly in RTECs of ADR-treated mice, and inhibition of CaMKII activity prevented this change (Figure 3(F)). Taken together, our data show that inhibition of CaMKII activity prevented the ADR-induced decrease in YAP expression both *in vitro* and *in vivo*. Thus, these data demonstrated that CaMKII regulates YAP.

### 3.4. Inhibition of CaMKII activity prevented ADR-induced nuclear NFAT2 increase

Our previous study [15] showed that NFAT2 expression is remarkably increased in ADR-injured podocytes. Thus, in this study, we examined NFAT2 expression in ADR-injured RTECs *in vitro* and *in vivo* to explore the underlying mechanism of NFAT2 in RTEC apoptosis. We used confocal microscopy to examine the localization of NFAT2 and western blotting to analyze the nuclear NFAT2 protein expression. As shown in Figure 3(A–C), the results of immunofluorescence analysis and western blotting indicate that the nuclear protein expression of NFAT2 was markedly increased in ADR-injured cultured RTECs. However, inhibition of CaMKII activity using K93 prevented ADR-induced increase in NFAT2 expression. In addition, the western blotting results show that the nuclear protein expression of NFAT2 increased significantly in kidney tissue cells of ADR-treated mice (*p* < 0.05), and inhibition of CaMKII activity prevented this change (*p* < 0.05; Figure 3(D–E)). Furthermore, the immunohistochemical staining results indicate that nuclear NFAT2 increased significantly in RTECs of ADR-treated mice, and inhibition of CaMKII activity prevented this change (Figure 3(F)). Thus, these data show that CaMKII regulates NFAT2.

### 3.5. CaMKII may regulate RTEC apoptosis through YAP

**Figure 4.**
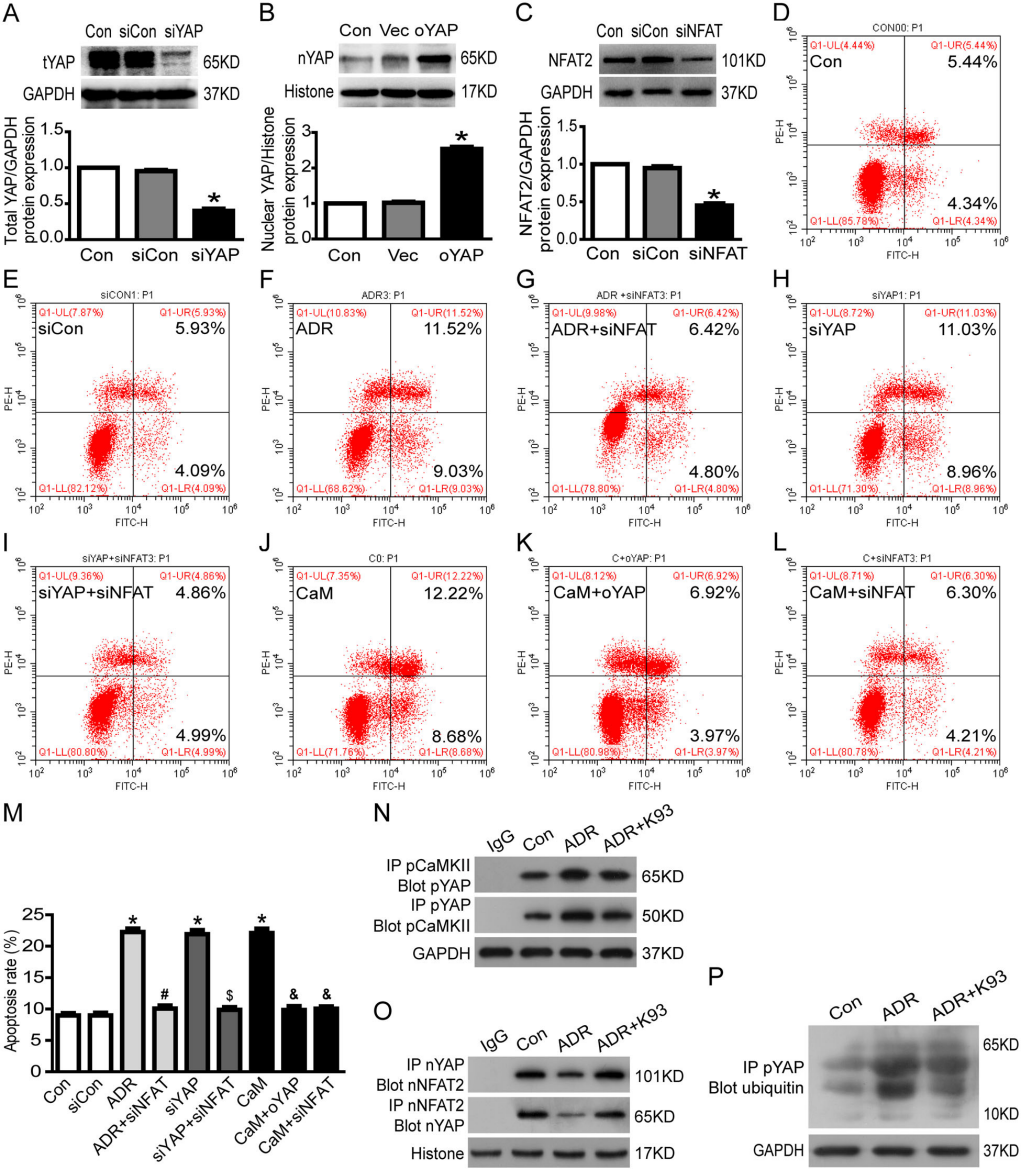
CaMKII may regulate RTEC apoptosis through YAP/NFAT2. (A) Total YAP protein expression was reduced to approximately 40.0% in YAP-siRNA-treated culture RTECs (*p* < 0.05). (B) In cultured RTECs, nuclear YAP protein expression increased to approximately 253.7% in the oYAP (overexpressed YAP plasmids) group (*p* < 0.05). (C) Total NFAT2 protein expression decreased to approximately 45.0% in NFAT2-siRNA-treated cultured RTECs (*p* < 0.05). (D–F, H, J) RTECs were stained with Annexin V/PI for flow cytometry analysis. The cell apoptosis rate increased significantly in ADR-, siYAP (YAP-siRNA)-, or CaM (CaMKII activator)-treated RTECs compared with that in the Con (controls) or siCon (blank siRNA control) groups (*p* < 0.05). (G, I, K-M) Cell apoptosis was significantly alleviated by using NFAT2-siRNA in ADR-, YAP-siRNA-, or CaM-treated RTECs (*p* < 0.05). Cell apoptosis was significantly alleviated by overexpressing YAP in CaM-injured RTECs (*p* < 0.05). (N) Co-immunoprecipitation results showing that cytoplasmic protein pCaMKII (phosphorylated CaMKII) and pYAP (phosphorylated YAP) were bound to each other. (O) Co-immunoprecipitation results showing that the nuclear proteins YAP (dephosphorylated YAP) and NFAT2 were bound to each other. (P) Ubiquitinated pYAP in the cytoplasm was detected using immunoprecipitation and western blot analysis. Mean ± SE, *n* = 6. *p < 0.05 vs. Con or siCon. #*p* < 0.05 vs. ADR. $*p* < 0.05 vs. siYAP. &*p* < 0.05 vs. CaM. There was no statistically significant difference among the Con, siCon, ADR + siNFAT, siYAP + siNFAT, CaM + oYAP, and CaM + siNFAT groups.

To further confirm CaMKII-regulated RTEC apoptosis through YAP, we used an annexin V/PI staining assay and immunoprecipitation to explore the underlying connection between CaMKII and YAP. Figure 4(D–E, H, J–K, M) shows that the cell apoptosis rate increased significantly in CaM (CaMKII activator)-injured or YAP-siRNA (YAP protein expression was knockdown to ∼40%, Figure 4(A))-treated RTECs compared with that in the Con (controls) or siCon (siRNA blank control) groups (*p* < 0.05), and cell apoptosis was significantly alleviated by overexpressing YAP (nuclear YAP protein overexpressed to approximately 253.7%, Figure 4(B)) in CaM-injured RTECs (*p* < 0.05). Furthermore, the results of co-immunoprecipitation showed that pCaMKII and pYAP bound to each other in the cytoplasm, and ADR accelerated the binding effect of pCaMKII and pYAP, whereas inhibition of CaMKII activity using K93 prevented the binding effect (Figure 4(N)). Immunoprecipitation and western blot analysis further showed that sequestrated pYAP in the cytoplasm was degraded by ubiquitination, and ADR seemed to increase the pYAP ubiquitinating effect, whereas inhibition of CaMKII activity using K93 prevented this effect (Figure 4(P)). Collectively, these data demonstrate that CaMKII induces RTEC apoptosis *via* YAP.

### 3.6. CaMKII/YAP may regulate RTEC apoptosis through NFAT2

We used an annexin V/PI staining assay and immunoprecipitation to further explore the underlying connection of CaMKII, YAP, and NFAT2. As shown in Figure 4(D–J, L–M), the cell apoptosis rate increased significantly in ADR-, YAP-siRNA-, and CaM (CaMKII activator)-treated RTECs compared with that in the Con (controls) or siCon (blank siRNA control) groups (*p* < 0.05). Cell apoptosis was significantly alleviated by using NFAT2-siRNA (NFAT2 protein expression was knocked down to ∼45%, Figure 4(C)) in ADR-, YAP-siRNA-, and CaM-treated RTECs (*p* < 0.05). Furthermore, the immunofluorescent staining (Figure 3(A)) and co-immunoprecipitation (Figure 4(O)) results show that the nuclear protein YAP (dephosphorylated YAP) and NFAT2 were bound to each other. ADR seemed to reduce the binding of YAP and NFAT2, whereas K93 accelerated the binding (Figure 4(O)). Collectively, these data suggest that CaMKII/YAP might regulate RTEC apoptosis *via* NFAT2.

## 4. Discussion

AKI is a global disease with high incidence and mortality. Epidemiological studies have revealed the huge medical and economic burden of AKI in Eastern Asian countries [24,25]. Preventive opportunities are missed because of the failure to recognize risk factors and early signs of AKI^24^. Furthermore, the underlying mechanisms that cause AKI are unclear, and treatment is insufficient. Apoptosis of RTEC is one of the most important events in AKI. This study explored the underlying mechanisms of CaMKII, YAP, and NFAT2 in RTEC apoptosis, implicating CaMKII as a potential target for ameliorating RTEC apoptosis in AKI.

CaMKII is a ubiquitously expressed serine/threonine kinase that plays an important role in diabetic nephropathy [26] and polycystic kidney diseases [5]. Several studies have shown that CaMKII activation is involved in cell death, including RTECs [4,7,27]. Consistent with these previous studies, we found that CaMKII activity was significantly increased in ADR-treated RTECs *in vitro* and *in vivo*. Activation of CaMKII-induced RTEC apoptosis *in vitro*. Both cultured RTEC and kidney tissue cell apoptosis induced by ADR were significantly ameliorated *via* inhibiting CaMKII activity. These data demonstrate that CaMKII plays an important role in RTEC apoptosis, both *in vitro* and *in vivo*.

NFAT2 plays an important role in apoptosis in kidney diseases [28]. The expression of NFAT2 increased significantly in RTECs of patients with severe renal fibrosis. Inhibition of the nuclear translocation of NFAT2 markedly suppresses TGFβ-induced HK-2 cell apoptosis *in vitro* [29]. Knockdown of CaMKII expression using siRNA inhibited Ang II-induced activation of NFAT2 nuclear transfer [30]. In addition, CaMK can promote NFAT function [10,31]. These findings indicate that CaMKII and NFAT2 may participate in the pathogenesis of RTEC apoptosis. In this study, we found that the nuclear protein expression of NFAT2 was markedly increased in ADR-injured cultured RTECs or kidney tissue cells of ADR-treated mice. Inhibition of CaMKII activity prevented the ADR-induced increase in NFAT2 expression both *in vitro* and *in vivo*. NFAT2-siRNA significantly alleviated ADR- or CaM-induced RTEC apoptosis. Thus, our data show that CaMKII may regulate RTEC apoptosis through NFAT2.

YAP is an apoptosis-antagonizing transcriptional co-activator [11,22]. Our previous studies showed that nuclear and cytoplasmic YAP levels are remarkably reduced in ADR-injured podocytes [16,17]. However, the reason for this decrease in nuclear and cytoplasmic YAP levels remained unclear. In this study, we showed that nuclear YAP is remarkably reduced in ADR-injured RTECs, pCaMKII and pYAP bond to each other in the cytoplasm, and sequestrated pYAP in the cytoplasm is degraded by ubiquitination. ADR could accelerate the binding effect of pCaMKII and pYAP and seemed to increase the ubiquitinating effect for pYAP. However, inhibition of CaMKII activity prevented these effects. Thus, these data show that CaMKII regulates YAP.

Our previous study found that YAP could bind to NFAT2 in the nucleus, thereby inhibiting NFAT-mediated podocyte apoptosis [15], and indicated that CaMKII/NFAT2 and YAP may together regulate RTEC apoptosis. In this study, we showed that apoptosis is significantly increased in YAP-siRNA-treated RTECs. CaMKII activator-induced RTEC apoptosis is alleviated by YAP overexpression. Apoptosis is significantly alleviated by using NFAT2-siRNA in ADR-, YAP-siRNA-, or CaMKII-activated RTECs. Inhibition of CaMKII activity prevents ADR-induced decrease in YAP expression *in vitro* and *in vivo*. Immunofluorescent staining and co-immunoprecipitation results show that the nuclear proteins YAP and NFAT2 are bound to each other. ADR reduces the binding effect of YAP and NFAT2, whereas inhibition of CaMKII activity reverses the binding effect. Collectively, these data indicate that CaMKII may regulate RTEC apoptosis *via* YAP/NFAT2.

## 5. Conclusion

**Figure 5.**
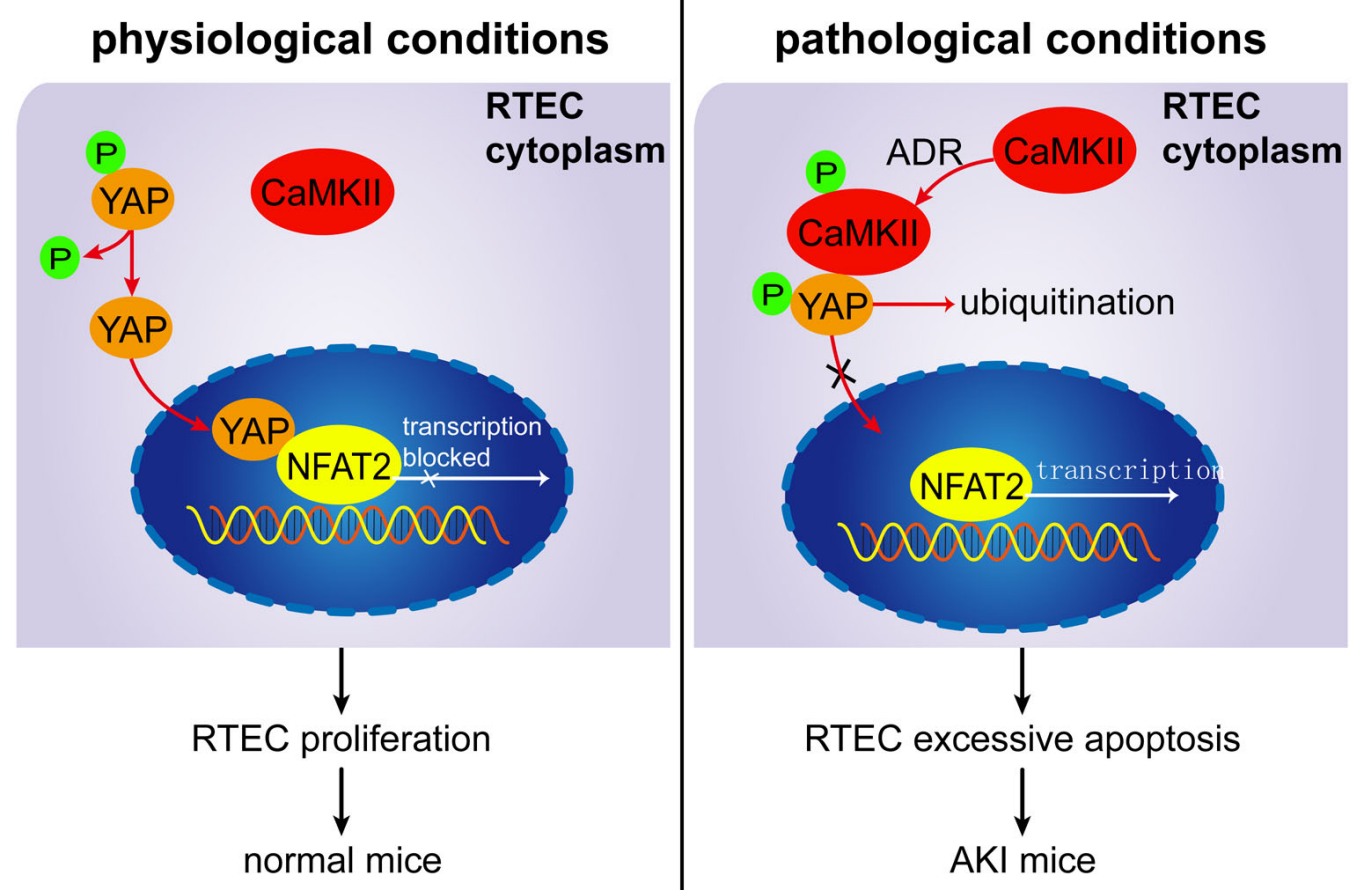
Proposed mechanism for CaMKII/YAP/NFAT regulating RTEC apoptosis in AKI mice. Under physiological conditions, dephosphorylated YAP is located at the RTEC nucleus and binds to NFAT to inhibit RTEC apoptosis. Under pathological conditions (ADR injury), pCaMKII increases and binds to pYAP in the cytoplasm, blocking pYAP from entering the nucleus and thereby preventing inhibition of NFAT2-mediated RTEC apoptosis. Sequestrated pYAP in the RTEC cytoplasm is degraded by ubiquitination. Excessive RTEC apoptosis induces AKI in ADR-treated mice.

In conclusion, this study found that CaMKII is involved in RTEC apoptosis. Under pathological conditions (ADR injury), pCaMKII is increased and can bind to pYAP in the cytoplasm, blocking pYAP from entering the nucleus and thereby preventing inhibition of NFAT2-mediated RTEC apoptosis. Sequestrated pYAP in RTEC cytoplasm is degraded by ubiquitination. Excessive RTEC apoptosis induces AKI in ADR-treated mice (Figure 5). Thus, our data demonstrate that CaMKII promotes RTEC apoptosis through YAP/NFAT2 and plays an important role in AKI, implicating that CaMKII may be a potent molecular target in AKI treatment.
